# Spontaneous pneumomediastinum in a term neonate – case report

**DOI:** 10.1002/ccr3.1352

**Published:** 2017-12-22

**Authors:** Gustavo Rocha, Hercília Guimarães

**Affiliations:** ^1^ Department of Neonatology Centro Hospitalar São João Porto Portugal; ^2^ Faculty of Medicine University of Porto Porto Portugal

**Keywords:** Air leak, cesarean, newborn, pneumomediastinum

## Abstract

Pneumomediastinum after birth, without birth injury or resuscitation maneuvers, is an unusual situation that can present with grunting, deafening of cardiac sounds, and bulging of the hemithorax. If clinical condition allows, conservative approach is advised even if the pneumomediastinum does not spontaneously resolve in 1 or 2 days.

## Introduction

Significant spontaneous pneumomediastinum and pneumothorax after birth in a term newborn without birth injury or resuscitation maneuvers are not frequent. Mild spontaneous pneumomediastinum after birth is relatively common and resolve in the first 24 h of life. Most cases are associated with prematurity, pneumonia, meconium aspiration, difficult delivery, and need for positive pressure during resuscitation or mechanical ventilation 1, 2, 3, 4, 5, 6.

We report the clinical case of a term newborn with a significant spontaneous pneumomediastinum after uncomplicated cesarean section, and who did not need resuscitation maneuvers, as an unusual presentation.

## Case Report

A 39 weeks gestational age Caucasian male newborn, with a birth weight of 3260 g, was born after a cesarean section because of pelvis presentation, to a 37‐years‐old primigravida with a followed and uneventful pregnancy. The Apgar score was 9 and 10 at first and fifth minutes, respectively, and there was no need for any resuscitation maneuver, namely positive pressure with bag and mask or T‐piece.

The neonate looked well and the first examination performed by a neonatologist after birth was normal. He went to the well baby nursery with the mother, in the obstetrics department. Two hours later, he developed a persistent grunting and showed also a poor suckling reflex. At four hours of life, the baby presented with persistent grunting, deafening of cardiac sounds, and bulging of the left hemithorax without subcutaneous emphysema.

**Figure 1:**
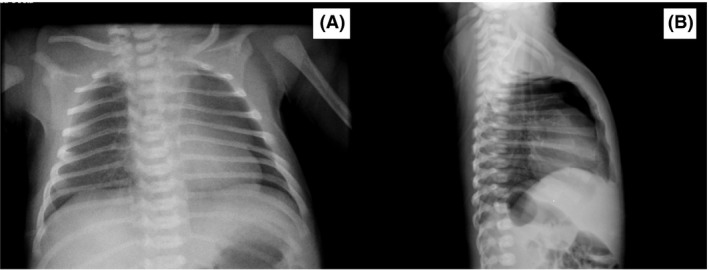
(A) Anterior pneumomediastinum on left hemithorax; (B) Lateral view of the pneumomediastinum.

The chest x‐ray film revealed a pneumomediastinum image (Fig. 1) and the arterial blood gases analysis was normal (pH 7.38; PO_2_ 62 mmHg; PCO_2_ 39 mmHg; Bic 21 mEq/L; BE 4 mEq/L; Sat 94%) and the pulse oximetry revealed a saturation of 99–100%. Grunting stopped in a few hours and the suckling reflex increased, but the pneumomediastinum image was still present in the chest x‐ray film performed on the two following days. The neonate was admitted to the neonatal intensive care unit on day three of life, after mother′s discharge, for clinical, image, and peripheral oxygen saturations monitoring. He was empirically started on 25% oxygen in the incubator to aid the reabsorption of the pneumomediastinum, which was maintained until day 9 of life. Blood cells count was within the normal range and blood cultures were sterile. Cranial, abdominal, and kidney ultrasound studies showed no abnormalities.

Daily chest x‐ray images revealed complete resorption of the pneumomediastinum by day nine of life, and the neonate was discharged on that day. He was examined at 2 months old at the outpatient department and was clinically well.

## Discussion

Neonatal pneumomediastinum occurs in approximately 2.5 per 1000 live births 7. According to the results of a study in 1971 by Steele RW and coworkers, in which chest films were obtained within two hours after birth in 300 routine vaginal deliveries, 100 premature infants, 100 infants born by cesarean section, and 50 intubated infants, the incidence of pneumomediastinum was demonstrated in 2.3% of normal deliveries, 2% of premature infants, 1% of cesarean sections, and 8% of intubated neonates. Apparent predisposing or associated factors included intubation, congenital anomalies, and meconium‐stained amniotic fluid. No significant correlation with prematurity, dysmaturity, small size for age, cesarean section, or hyaline membrane disease was found 7.

Spontaneous neonatal pneumomediastinum is the result of air leak due to increases in alveolar pressure and is not common after uncomplicated deliveries 1. In these cases, the pressure gradient between the alveolar and perivascular space could elevate abnormally high during crying of the baby and an alveolar rupture may occur. The infant is often asymptomatic, but respiratory distress or persistent grunting may be the clinical signs 8.

C‐section delivery has been associated with increased respiratory distress in newborns and high transpulmonary pressure may lead to a rupture of the lung and pneumomediastinum if there is a bronchial obstruction from liquid or mucus 9.

Radiographically, a pneumomediastinum may present in several ways. The classic description is that of a “wind‐blown spinnaker sail” (a lobe or lobes of the thymus being elevated off the heart), most likely to be seen on a left lateral oblique view. In other cases, a halo may be seen around the heart in the anteroposterior projection. This must be distinguished from a pneumopericardium in which air completely surrounds the heart, including the inferior border. The cross‐table lateral projection will show an anterior collection of air that may be difficult to distinguish from a pneumothorax, as in the reported case.

Close observation and monitoring of the patient may be the only measure needed, although some cases may need drainage and/or mechanical ventilation, including high‐frequency oscillatory ventilation, depending on the severity of the underlying respiratory distress and the degree of compromise caused by the air leak. One should resist the temptation to insert a drain into the mediastinum because it will not be beneficial and may cause more problems than it will solve. An oxygen‐rich environment can be used in the term infant to attempt nitrogen washout if the pneumomediastinum is believed to be clinically significant 10.

Conservative treatment and close follow‐up until resolution are suggested because of the potential risk of pneumothorax, subcutaneous, and interstitial emphysema with deterioration of the clinical status.

## Authorship

GR: written the clinical report and HG: reviewed the case report.

## Conflict of Interest

None declared.
